# The association between dietary antioxidant quality score and uric acid related mortality in patients with chronic kidney disease

**DOI:** 10.3389/fnut.2024.1408898

**Published:** 2024-07-12

**Authors:** Shuai Shi, Qiang Fang

**Affiliations:** ^1^Department of Rheumatic Nephrology, The Sixth Clinical Medical College of Xinjiang Medical University, Ürümqi, China; ^2^Department of Nephrology, The Affiliated Taizhou People's Hospital of Nanjing Medical University, Taizhou, China

**Keywords:** chronic kidney disease, antioxidant dietary, hyperuricemia, mortality, NHANES

## Abstract

**Aim:**

Antioxidants diet is beneficial for the prognosis of chronic kidney disease (CKD). However, the relationship between the Dietary Antioxidant Quality Score (DAQS), a measure of overall quality on antioxidant diet, and hyperuricemia related mortality is unclear. This study aimed to investigate the relationship between the DAQS and hyperuricemia mortality in CKD patients.

**Methods:**

In this cohort study, data were collected in the National Health and Nutrition Examination Survey (NHANES) from 2009 to 2018. The DAQS was calculated based on the six dietary antioxidants. Mortality status were determined by NHANES-linked National Death Index public access files through December 31, 2019. Weighted Cox proportional hazard models were used to investigate the association between the DAQS and hyperuricemia related mortality.

**Results:**

A total of 3,684 participants were included. During the median follow-up of 63.83 months, 820 deaths were recorded. The results showed that higher dietary antioxidants intake associated with lower hyperuricemia related mortality risk among CKD patients (HR = 1.28, 95%CI: 1.07 to 1.54). In subgroup analyses, the association of antioxidants intake and hyperuricemia related mortality risk remained exist in groups of aged ≥65 years (HR = 1.23, 95%CI: 1.01 to 1.52), with hypertension (HR = 1.26, 95%CI: 1.02 to 1.55), with dyslipidemia (HR = 1.30, 95%CI: 1.07 to 1.58), with CVD (HR = 1.31, 95%CI: 1.03 to 1.67), and diabetes (HR = 1.62, 95%CI: 1.24 to 2.12).

**Conclusion:**

Higher antioxidants intake associated with lower odds of hyperuricemia related mortality in CKD patients. Future interventional studies are needed to elucidate the beneficial effect of antioxidants diets.

## Introduction

Chronic kidney disease (CKD), characterized by the progressive renal function decline, is a global health problem affecting millions of individuals worldwide (1). In the United States, CKD affects 37 million adults (2). CKD has continued to rise in rank among leading cause of mortality with 1.2 million global deaths attributed to CKD in 2017 (1). The globally all-age CKD mortality rate has increased by 41.5% from 1999 to 2017 (1). Therefore, accurately identifying factors affecting the prognosis of CKD is crucial for implementing reasonable intervention and reducing the disease burden.

Uric acid (UA), as an end product of purine metabolism in humans, has emerged as a potential risk factor for adverse outcomes in CKD (3). Elevated UA levels are associated with increased oxidative stress (OS) and inflammation, both of which plays a crucial role in CKD progression (4). Increased serum UA levels are associated with higher risk of all-cause and cardiovascular disease (CVD) mortality among CKD patients (5, 6). A review reported that the primary benefit of lowering serum urate is by reducing the incidence of cardiovascular events and mortality in CKD (7). Therefore, identifying strategies to mitigate the detrimental effects of UA is of paramount importance.

Medical nutrition therapy is essential for CKD patients as it can slow disease progression (2). Dietary antioxidants, which can neutralize harmful reactive oxygen species (ROS) and protect against cellular damage, have gained considerable attention for their ability to counteract OS and mitigate inflammation (8–10). An antioxidant-rich diet may confer protective effects against CKD development and progression, while moderate dietary antioxidants intake is linked to reduced mortality risk in early-stage CKD patients (11–13). The Dietary Antioxidant Quality Score (DAQS) is a comprehensive measure that assesses the overall quality of antioxidant intake from dietary sources. The DAQS considers various antioxidants including vitamin A, vitamin C, vitamin E, zinc, magnesium, and selenium (14), providing a quantitative assessment of antioxidant intake. The DAQS has been used to evaluate the association between antioxidant intake and various health outcomes, such as diabetes (14), metabolic syndrome (15), and systemic lupus erythematosus (16). However, the relationship between dietary antioxidant intake and hyperuricemia-related mortality in CKD remains unknown. Therefore, this study aims to investigate the association of dietary antioxidant intake, hyperuricemia, with mortality in CKD patients and further explore the ameliorative effect of antioxidant intake on the relationship between hyperuricemia and all-cause mortality.

## Methods

### Study design and participants

The study population of this cohort study were extracted from the National Health and Nutrition Examination Surveys (NHANES) (2009–2018). NHANES, major program of the National Center for Health Statistics (NCHS), is designed to assess the health and nutritional status of adults and children in the United States, with combined interviews and physical examinations.

Participants with CKD were included from the database. CKD was defined as urinary albumin to creatinine ratio (UACR) >30 mg/g and/or estimated glomerular filtration rate (eGFR) <60 mL/min/1.73m^2^ according to the “KDIGO 2021 Guidelines” (17). Urinary albumin was measured by solid-phase fluorescent immunoassay. And eGFR was calculated by using the Chronic Kidney Disease Epidemiology Collaboration (CKD-EPI) equation for standardized creatinine (18). The equation is eGFR (mL/min/1.73m^2^) = 141 × min (Scr/κ, 1)^α^ × max (Scr/κ, 1)^-1.209^ × 0.933^age^ × 1.108 (if female) × 1.159 (if black). κ is 0.7 for females and 0.9 for males, α is −0.329 for females and − 0.411 for males, min indicates the minimum of Scr/κ or 1, and max indicates the maximum of Scr/κ or 1. Exclusion criteria were as follows: (1) <18 years old, (2) missing data on uric acid, (3) missing data on energy intake, (4) with implausible energy intake (<500 kcal or > 8,000 kcal in male or < 500 kcal or > 5,000 kcal in female), and (5) missing survival information. The NHANES protocol was approved by the NCHS Research Ethics Review Board and all participants signed an informed consent.

### Assessment of uric acid

UA in serum was measured by using a timed endpoint method based on Beckman Coulter UniCel® DxC800 (19). Hyperuricemia was defined as serum UA level > 7.0 mg/dL in males and > 6.0 mg/dL in females (20).

### Assessment of the DAQS

The DAQS were calculated based on six antioxidant vitamins and minerals, including vitamin A, C, E, zinc, magnesium, and selenium. A 24-h dietary interview was conducted by trained interviewers to collect data on dietary intake of six dietary antioxidant micronutrients. The daily intakes for each antioxidant were calculated as the sum of dietary and supplement intake. For the DAQS, daily nutrient intake of each of six nutrients/minerals were compared with their respective daily recommended intake (RDI) as determined by the Dietary Guidelines for Americans 2015–2020 (14). Then, each antioxidant vitamin/mineral was assigned a value of either 0 or 1, that 0 defined as intake of <2/3 of the RDI and 1 defined as intake ≥2/3 of the RDI. The summed DAQS ranged from 0 (very poor quality) to 6 (high quality). Then, the DAQS was classified into two groups: 1–4 (low quality) and 5–6 (high quality).

### Covariates

Potential covariates were considered in this study. Included covariates were as follows: age, gender, race, marital status, poverty income ratio (PIR), smoking, CVD, diabetes, hemoglobin A1c (HbA1c), alkaline phosphatase (ALP), and asparate aminotransferase (AST). Information on age, gender, race, marital status, PIR, smoking, disease status and medication use was collected from household interviews using standardized questionnaires. Smoking was defined as participants who had a positive answer to the question “Smoked at least 100 cigarettes in life” (21). CVD was determined by a combination of self-reported physician diagnoses and cardiovascular medication usage. Diabetes was defined as meeting any of the following criteria: self-report of a diagnosis by a doctor or other health care professional, HbA1c ≥6.5% or fasting plasma glucose ≥7.0 mmol/L, and taking hypoglycemic medications and/or insulin (22). In addition, HbA1c, ALT and AST were measured when participants provided their blood samples. Details about procedure of blood collection and analysis were described in the NHANES Laboratory/Medical Technologists Procedures Manual (23).

### Outcomes and follow-up

The outcome of our research was all-cause mortality, defined as death from any cause. All-cause mortality was extracted from the National Death Index (NDI) database of the Centers for Disease Control through December 31, 2019. All data in this study were available.^1^ Follow-up time was defined from the data of participation to the data of death on December 31, 2019, whichever came first.

### Statistical analysis

Data were analyzed based on the prescribed guidelines for analysis of complex NHANES data set, taking into account the masked variance and utilizing the proposed weighting methodology (24). Continuous variables were presented as mean and standard error (S.E), while categorical variables were presented as frequency and percentage (%). Groups different among continuous and categorical variables were compared using the weighted t tests and chi-square tests, respectively. Confounders were selected for variables with statistical differences using a weighted univariate Cox proportional hazard model. The association between DAQS and UA related mortality was analyzed by weighted univariate and multivariable Cox proportional hazard models. Covariates were adjusted for age, gender, race, marital status, PIR, smoking, CVD, diabetes, HbA1c, ALP, and AST. Subgroup analyses were performed to further investigate the association between DAQS and UA related mortality in groups among age, hypertension, dyslipidemia, CVD, diabetes and CKD stage. Furthermore, imputations were performed for missing variables. *p* < 0.05 was considered statistically significant. All statistical analyses were conducted by using SAS 9.4 (SAS Institute Inc., Cary, NC, United States) and R software (version 4.2.2), while missing variates were performed by Python (version 3.9.12).

## Results

### Characteristics of participants

**Figure 1:**
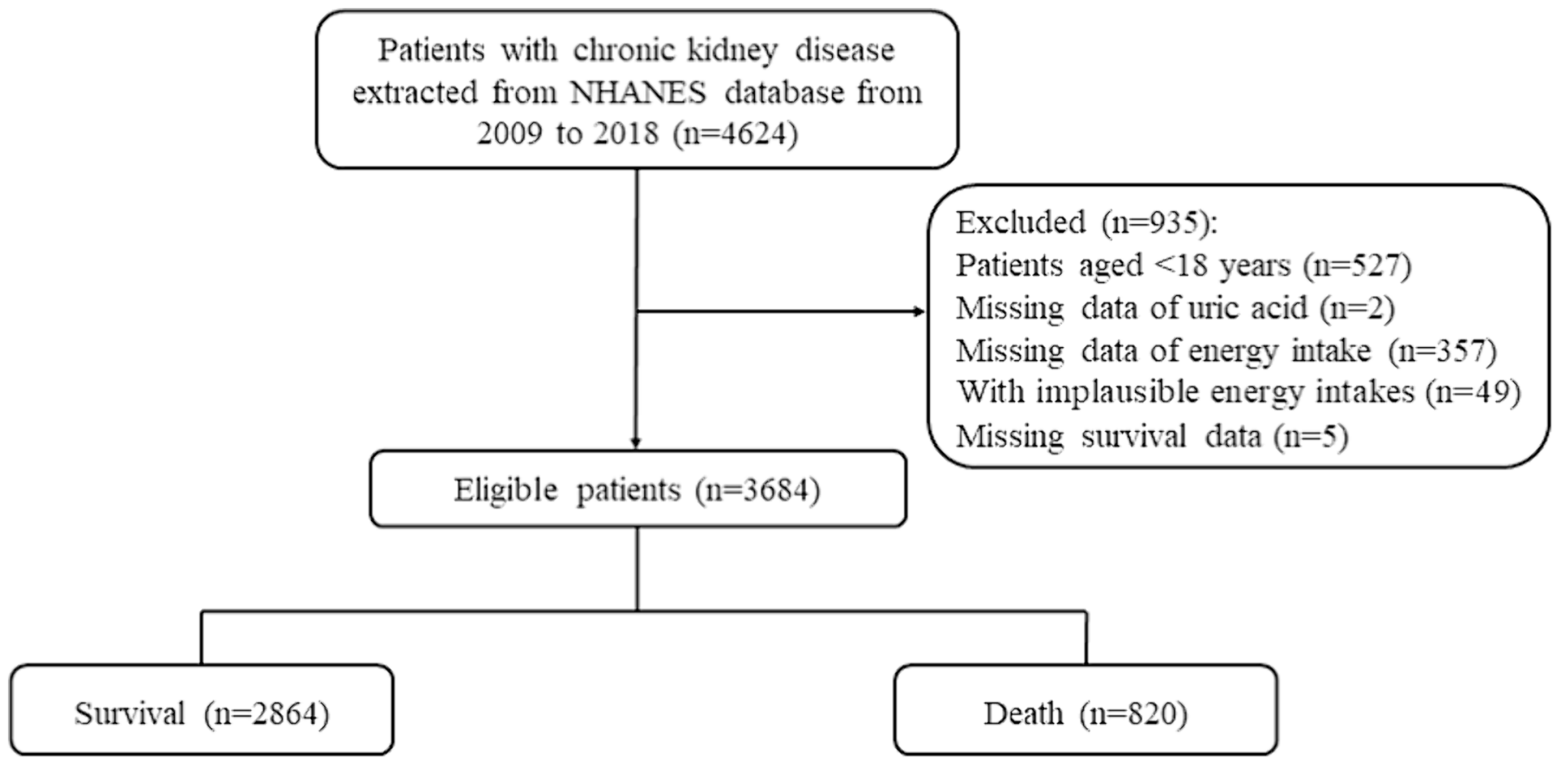
Flowing chart showing the selection of study participants.

In total, 4,624 participants in database from 2009 to 2018 were CKD. First, individuals were excluded with aged younger than 18 years (*n* = 527) and without uric acid information (*n* = 2), and total number of people was 4,095. In addition, individuals without energy intake information (*n* = 357) and with implausible energy intake (*n* = 49). Then, individuals missing survival information were excluded (*n* = 5). Finally, 3,684 participants were enrolled in the final analysis. Figure 1 shows the flow diagram of participants selection. After imputation, significant difference was not observed among missing values (Supplementary Table S1). A total of 820 deaths were identified during a follow-up period of 63.83 months. And 21 deaths due to renal disease. As shown in Table 1, the mean age was 58.94 years in this population. Among the group of alive, participants had higher education level, more physical exercises, less drinkers, and less comorbidities including CVD, diabetes and cancer. Participants dead were composed of more people who were older, who had high UA level, who were severe or end stage of CKD, and who were dyslipidemia and hypertension comorbidities.

**Table 1:** Characteristics of participants with CKD.

Variables	Total (*n* = 3,684)	Survival status			
		Survival (*n* = 2,864)	Death (*n* = 820)	Statistics	*p*
Age, years, Mean (S.E)	58.94 (0.45)	55.93 (0.50)	71.56 (0.46)	*t* = −23.51	<0.001
Age, *n* (%)				χ^2^ = 174.972	<0.001
<65	1806 (53.05)	1,653 (60.17)	153 (23.15)		
≥65	1878 (46.95)	1,211 (39.83)	667 (76.85)		
Gender, *n* (%)				χ^2^ = 6.465	0.011
Female	1860 (54.01)	1,517 (55.21)	343 (48.99)		
Male	1824 (45.99)	1,347 (44.79)	477 (51.01)		
Race, *n* (%)				χ^2^ = 53.676	<0.001
White	1,611 (66.43)	1,105 (63.54)	506 (78.59)		
Black	831 (12.45)	680 (13.13)	151 (9.62)		
Others	1,242 (21.12)	1,079 (23.34)	163 (11.79)		
Education level, *n* (%)				χ^2^ = 40.185	<0.001
High school graduate or below	1937 (45.28)	1,441 (43.44)	496 (53.00)		
Some college or above	1,655 (52.73)	1,336 (54.17)	319 (46.67)		
Unknown	92 (1.99)	87 (2.39)	5 (0.33)		
Marital status, *n* (%)				χ^2^ = 18.932	<0.001
Married	1782 (51.76)	1,402 (52.36)	380 (49.28)		
No married	1812 (46.29)	1,376 (45.30)	436 (50.43)		
Unknown	90 (1.95)	86 (2.35)	4 (0.29)		
PIR, ratio, Mean (S.E)	2.63 (0.05)	2.69 (0.06)	2.34 (0.07)	*t* = 4.52	<0.001
Smoking, *n* (%)				χ^2^ = 28.816	<0.001
Yes	1774 (48.64)	1,292 (46.47)	482 (57.77)		
No	1863 (50.32)	1,530 (52.35)	333 (41.79)		
Unknown	47 (1.04)	42 (1.18)	5 (0.44)		
Drinking, *n* (%)				χ^2^ = 3.717	0.156
Excessive drinking	327 (11.31)	264 (12.00)	63 (8.44)		
Light drinking	292 (9.21)	226 (9.32)	66 (8.76)		
Never drinking	3,065 (79.48)	2,374 (78.69)	691 (82.80)		
Physical activity, *n* (%)				χ^2^ = 131.421	<0.001
<450	1,037 (29.13)	832 (30.11)	205 (25.01)		
≥450	1,223 (36.45)	1,077 (40.70)	146 (18.61)		
Unknown	1,424 (34.42)	955 (29.19)	469 (56.38)		
Hypertension, *n* (%)				χ^2^ = 77.083	<0.001
No	672 (22.25)	612 (25.90)	60 (6.94)		
Yes	3,012 (77.75)	2,252 (74.10)	760 (93.06)		
Dyslipidemia, *n* (%)				χ^2^ = 27.330	<0.001
No	690 (19.51)	575 (21.24)	115 (12.25)		
Yes	2,994 (80.49)	2,289 (78.76)	705 (87.75)		
CVD, *n* (%)				χ^2^ = 157.883	<0.001
No	2013 (59.00)	1749 (64.58)	264 (35.57)		
Yes	1,671 (41.00)	1,115 (35.42)	556 (64.43)		
Diabetes, *n* (%)				χ^2^ = 38.409	<0.001
No	2,203 (65.19)	1779 (67.78)	424 (54.31)		
Yes	1,481 (34.81)	1,085 (32.22)	396 (45.69)		
Cancer, *n* (%)				χ^2^ = 78.742	<0.001
Yes	614 (17.65)	395 (15.04)	219 (28.63)		
No	2,976 (80.34)	2,379 (82.55)	597 (71.08)		
Unknown	94 (2.01)	90 (2.42)	4 (0.29)		
BMI, kg/m^2^, Mean (S.E)	30.44 (0.19)	30.49 (0.21)	30.19 (0.34)	*t* = 0.79	0.434
BMI, *n* (%)				χ^2^ = 2.231	0.328
Obesity	1,676 (46.73)	1,347 (47.36)	329 (44.06)		
Overweight	1,166 (30.07)	888 (29.93)	278 (30.65)		
Underweight/normal	842 (23.20)	629 (22.71)	213 (25.29)		
eGFR, mL/min/1.73m^2^, Mean (S.E)	82.83 (0.65)	87.18 (0.76)	64.57 (1.19)	*t* = 15.22	<0.001
UACR, mg/g, Mean (S.E)	197.23 (12.66)	179.70 (13.15)	270.82 (33.88)	*t* = −2.54	0.013
WBC, 1000 cells/uL, Mean (S.E)	7.59 (0.06)	7.54 (0.06)	7.79 (0.15)	*t* = −1.56	0.124
Lymphocyte count, 1,000 cells/uL, Mean (S.E)	2.07 (0.03)	2.10 (0.03)	1.95 (0.09)	*t* = 1.69	0.095
Neutrophil count, 1,000 cells/uL, Mean (S.E)	4.65 (0.04)	4.59 (0.04)	4.91 (0.11)	*t* = −2.88	0.005
Platelet count, 1,000 cells/uL, Mean (S.E)	233.46 (1.84)	237.37 (2.16)	217.03 (3.87)	*t* = 4.52	<0.001
Hemoglobin, g/dL, Mean (S.E)	13.77 (0.05)	13.88 (0.05)	13.33 (0.09)	*t* = 5.79	<0.001
Uric acid, mg/dL, Mean (S.E)	5.92 (0.04)	5.80 (0.04)	6.41 (0.07)	*t* = −7.31	<0.001
ALT, U/L, Mean (S.E)	24.54 (0.55)	24.70 (0.49)	23.86 (1.88)	*t* = 0.44	0.662
ALP, U/L, Mean (S.E)	74.17 (0.67)	73.41 (0.73)	77.39 (1.43)	*t* = −2.56	0.012
AST, U/L, Mean (S.E)	26.43 (0.50)	25.71 (0.37)	29.43 (1.99)	*t* = −1.85	0.068
GGT, U/L, Mean (S.E)	33.36 (1.44)	31.06 (0.85)	43.05 (6.78)	*t* = −1.74	0.086
Energy, kcal, Mean (S.E)	1970.56 (19.83)	2012.36 (22.91)	1795.11 (31.39)	*t* = 5.80	<0.001
Protein, gm, Mean (S.E)	75.46 (0.93)	76.99 (0.98)	69.00 (1.86)	*t* = 4.13	<0.001
Carbohydrate, mg, Mean (S.E)	234.24 (2.61)	237.53 (2.93)	220.40 (3.99)	*t* = 3.73	<0.001
Total fat, mg, Mean (S.E)	76.70 (0.92)	78.73 (1.07)	68.19 (1.54)	*t* = 5.78	<0.001
Sodium, mg, Mean (S.E)	3274.40 (37.11)	3346.71 (41.21)	2970.86 (61.79)	*t* = 5.54	<0.001
Potassium, mg, Mean (S.E)	2554.77 (28.26)	2577.72 (31.54)	2458.42 (45.62)	*t* = 2.33	0.022
Calcium, mg, Mean (S.E)	1077.40 (13.72)	1073.48 (16.17)	1093.84 (22.66)	*t* = −0.73	0.469
Vitamin D, mcg, Mean (S.E)	24.54 (2.64)	23.84 (3.02)	27.48 (4.51)	*t* = −0.69	0.491
Vitamin A, mcg, Mean (S.E)	618.51 (11.24)	622.54 (13.00)	601.56 (23.13)	*t* = 0.78	0.440
Vitamin C, mg, Mean (S.E)	174.50 (6.62)	171.89 (7.33)	185.49 (12.24)	*t* = −1.00	0.318
Vitamin E, mg, Mean (S.E)	8.10 (0.13)	8.28 (0.15)	7.38 (0.24)	*t* = 3.01	0.004
Zinc, mg, Mean (S.E)	15.84 (0.34)	15.70 (0.36)	16.42 (0.65)	*t* = −1.07	0.288
Magnesium, mg, Mean (S.E)	312.93 (3.96)	317.66 (4.45)	293.07 (7.06)	*t* = 3.07	0.003
Selenium, mcg, Mean (S.E)	122.33 (1.88)	123.10 (1.79)	119.09 (5.13)	*t* = 0.78	0.437
CKD stage, *n* (%)				χ^2^ = 32.242	<0.001
Severe/end stage	3,569 (97.52)	2,799 (98.28)	770 (94.32)		
Mild/moderate	115 (2.48)	65 (1.72)	50 (5.68)		
DAQS, score, Mean (S.E)	3.76 (0.04)	3.77 (0.04)	3.70 (0.08)	*t* = 0.82	0.415
DAQS, *n* (%)				χ^2^ = 0.735	0.391
High	1,193 (37.11)	930 (37.58)	263 (35.16)		
Low	2,491 (62.89)	1934 (62.42)	557 (64.84)		
Hyperuricemia, *n* (%)				χ^2^ = 23.643	<0.001
No	2,463 (67.31)	1992 (69.55)	471 (57.90)		
Yes	1,221 (32.69)	872 (30.45)	349 (42.10)		
Follow time, months, Mean (S.E)	63.83 (1.15)	67.15 (1.33)	49.90 (1.50)	*t* = 9.55	<0.001
Survival status, *n* (%)					
Survival	2,864 (80.76)	2,864 (100.00)	0 (0.00)		
Death for renal disease	21 (0.50)	0 (0.00)	21 (2.62)		
Death for other causes	799 (18.74)	0 (0.00)	799 (97.39)		

t, *t* test; χ^2^, chi-square test; S.E, standard error. CKD, chronic kidney disease; PIR, poverty income ratio; CVD, cardiovascular disease; BMI, body mass index; eGFR, estimated glomerular filtration rate; UACR, urinary albumin to creatinine ratio; WBC, white blood count; ALT, alanine aminotransferase; ALP, alkaline phosphatase; AST, asparate aminotransferase; GGT, gamma-glutamyl transferase; DAQS, the dietary antioxidant quality score.

### Association of overall antioxidants intake, UA and mortality

The relation between UA and all-cause mortality was observed in Table 2. The risk of all-cause mortality was increased in the population with hyperuricemia (HR = 1.20, 95%CI: 1.01 to 1.41). Table 3 shows the association of overall antioxidants intake with UA related mortality. After adjusting age, gender, race, marital status, PIR, smoking, CVD, diabetes, hemoglobin, ALP, and AST, the lower DAQS was associated with increased risk of all-cause mortality in hyperuricemia population (HR = 1.28, 95%CI: 1.07 to 1.54). In participants with higher DAQS, the association of hyperuricemia with all-cause mortality was not found (HR = 1.07, 95%CI: 0.76 to 1.50). Higher antioxidants intake may ameliorate the risk of hyperuricemia related mortality.

**Table 2:** Association between DAQS, hyperuricemia and all-cause mortality in CKD patients.

Variables	Univariable model		Multivariable model^*^	
	HR (95%CI)	*p*	HR (95%CI)	*p*
DAQS				
High	Ref		Ref	
Low	1.04 (0.84–1.28)	0.739	0.98 (0.81–1.18)	0.805
Hyperuricemia				
No	Ref		Ref	
Yes	1.65 (1.39–1.96)	<0.001	1.20 (1.01–1.41)	0.039

Ref, reference; HR, hazard ratio; CI, Confidence interval; CKD, chronic kidney disease; DAQS, the dietary antioxidant quality score; PIR, poverty income ratio; CVD, cardiovascular disease; ALP, alkaline phosphatase; AST, asparate aminotransferase. ^*^Adjusted for age, gender, race, marital status, PIR, smoking, CVD, diabetes, hemoglobin, ALP, and AST.

**Table 3:** Associations between dietary antioxidants intake and hyperuricemia related mortality in CKD patients.

Variables	Univariable model		Multivariable model^*^	
	HR (95%CI)	*p*	HR (95%CI)	*p*
DAQS: High (*n* = 1,193)				
Hyperuricemia				
No	Ref		Ref	
Yes	1.43 (1.03–1.98)	0.033	1.07 (0.76–1.50)	0.706
DAQS: Low (n = 2,491)				
Hyperuricemia				
No	Ref		Ref	
Yes	1.77 (1.44–2.17)	<0.001	1.28 (1.07–1.54)	0.009

Ref, reference; HR, hazard ratio; CI, confidence interval; CKD, chronic kidney disease; DAQS, the Dietary Antioxidant Quality Score; PIR, poverty income ratio; CVD, cardiovascular disease; ALP, alkaline phosphatase; AST, asparate aminotransferase. ^*^Adjusted for age, gender, race, marital status, PIR, smoking, CVD, diabetes, hemoglobin, ALP, and AST.

### Subgroup analysis

In order to investigate whether patient characteristics and comorbidities could influence the association between DAQS and hyperuricemia related mortality, subgroup analyses were performed. Patients with lower DAQS were associated with hyperuricemia related mortality in subgroups. The association was observed among participants aged ≥65 years (HR = 1.23, 95%CI: 1.01 to 1.52), with hypertension (HR = 1.26, 95%CI: 1.02 to 1.55), with dyslipidemia (HR = 1.30, 95%CI: 1.07 to 1.58), with CVD (HR = 1.31, 95%CI: 1.03 to 1.67), and diabetes (HR = 1.62, 95%CI: 1.24 to 2.12). Notably, this association was observed in all stage of CKD (see Table 4).

**Table 4:** Association between DAQS and hyperuricemia related mortality in subgroups of age, hypertension, dyslipidemia, CVD, diabetes, and CKD stage.

Subgroups	Variables	HR (95%CI)^*^	*p*
Age < 65 (*n* = 1806)	DAQS: High (*n* = 560)		
	Hyperuricemia		
	No	Ref	
	Yes	1.31 (0.67–2.54)	0.428
	DAQS: Low (*n* = 1,246)		
	Hyperuricemia		
	No	Ref	
	Yes	1.00 (0.55–1.81)	1.000
Age ≥ 65 (*n* = 1878)	DAQS: High (*n* = 633)		
	Hyperuricemia		
	No	Ref	
	Yes	1.03 (0.71–1.49)	0.860
	DAQS: Low (*n* = 1,245)		
	Hyperuricemia		
	No	Ref	
	Yes	1.23 (1.01–1.52)	0.047
Hypertension = No (*n* = 672)	DAQS: High (*n* = 231)		
	Hyperuricemia		
	No	Ref	
	Yes	1.20 (0.29–4.98)	0.799
	DAQS: Low (*n* = 441)		
	Hyperuricemia		
	No	Ref	
	Yes	1.45 (0.61–3.43)	0.393
Hypertension = Yes (*n* = 3,012)	DAQS: High (*n* = 962)		
	Hyperuricemia		
	No	Ref	
	Yes	1.06 (0.75–1.50)	0.740
	DAQS: Low (*n* = 2050)		
	Hyperuricemia			
	No	Ref	
	Yes	1.26 (1.02–1.55)	0.029
Dyslipidemia = No (*n* = 690)	DAQS: High (*n* = 225)		
	Hyperuricemia		
	No	Ref	
	Yes	0.54 (0.25–1.16)	0.113
	DAQS: Low (*n* = 465)		
	Hyperuricemia		
	No	Ref	
	Yes	1.18 (0.66–2.10)	0.580
Dyslipidemia = Yes (*n* = 2,994)	DAQS: High (*n* = 968)		
	Hyperuricemia		
	No	Ref	
	Yes	1.05 (0.73–1.51)	0.773
	DAQS: Low (*n* = 2026)		
	Hyperuricemia		
	No	Ref	
	Yes	1.30 (1.07–1.58)	0.008
CVD=No (*n* = 2013)	DAQS: High (*n* = 668)		
	Hyperuricemia		
	No	Ref	
	Yes	0.92 (0.51–1.67)	0.788
	DAQS: Low (*n* = 1,345)		
	Hyperuricemia		
	No	Ref	
	Yes	1.24 (0.88–1.74)	0.212
CVD = Yes (*n* = 1,671)	DAQS: High (*n* = 525)		
	Hyperuricemia		
	No	Ref	
	Yes	1.12 (0.77–1.64)	0.549
	DAQS: Low (*n* = 1,146)		
	Hyperuricemia		
	No	Ref	
	Yes	1.31 (1.03–1.67)	0.027
Diabetes = No (*n* = 2,203)	DAQS: High (*n* = 747)		
	Hyperuricemia		
	No	Ref	
	Yes	1.01 (0.69–1.45)	0.982
	DAQS: Low (*n* = 1,456)		
	Hyperuricemia		
	No	Ref	
	Yes	1.03 (0.76–1.39)	0.861
Diabetes = Yes (*n* = 1,481)	DAQS: High (*n* = 446)		
	Hyperuricemia			
	No	Ref	
	Yes	1.15 (0.73–1.80)	0.538
	DAQS: Low (*n* = 1,035)		
	Hyperuricemia		
	No	Ref	
	Yes	1.62 (1.24–2.12)	<0.001
CKD stage = Severe/End stage (*n* = 3,569)	DAQS: High (*n* = 1,166)		
	Hyperuricemia		
	No	Ref	
	Yes	1.04 (0.73–1.50)	0.814
	DAQS: Low (*n* = 2,403)		
	Hyperuricemia		
	No	Ref	
	Yes	1.22 (1.01–1.47)	0.038
CKD stage = Mild/Moderate (*n* = 115)	DAQS: High (*n* = 27)		
	Hyperuricemia		
	No	Ref	
	Yes	2.89 (0.00–5166.84)	0.604
	DAQS: Low (*n* = 88)		
	Hyperuricemia		
	No	Ref	
	Yes	3.05 (1.45–6.45)	0.006

Ref, reference; HR, hazard ratio; CI, confidence interval; DAQS, the Dietary Antioxidant Quality Score; CKD, chronic kidney disease; CVD, cardiovascular disease; PIR, poverty income ratio; ALP, alkaline phosphatase; AST, asparate aminotransferase. Adjusted for age, gender, race, marital status, PIR, smoking, CVD, diabetes, hemoglobin, ALP, and AST. The corresponding confounding is removed to different subgroups.

## Discussion

**Figure 2:**
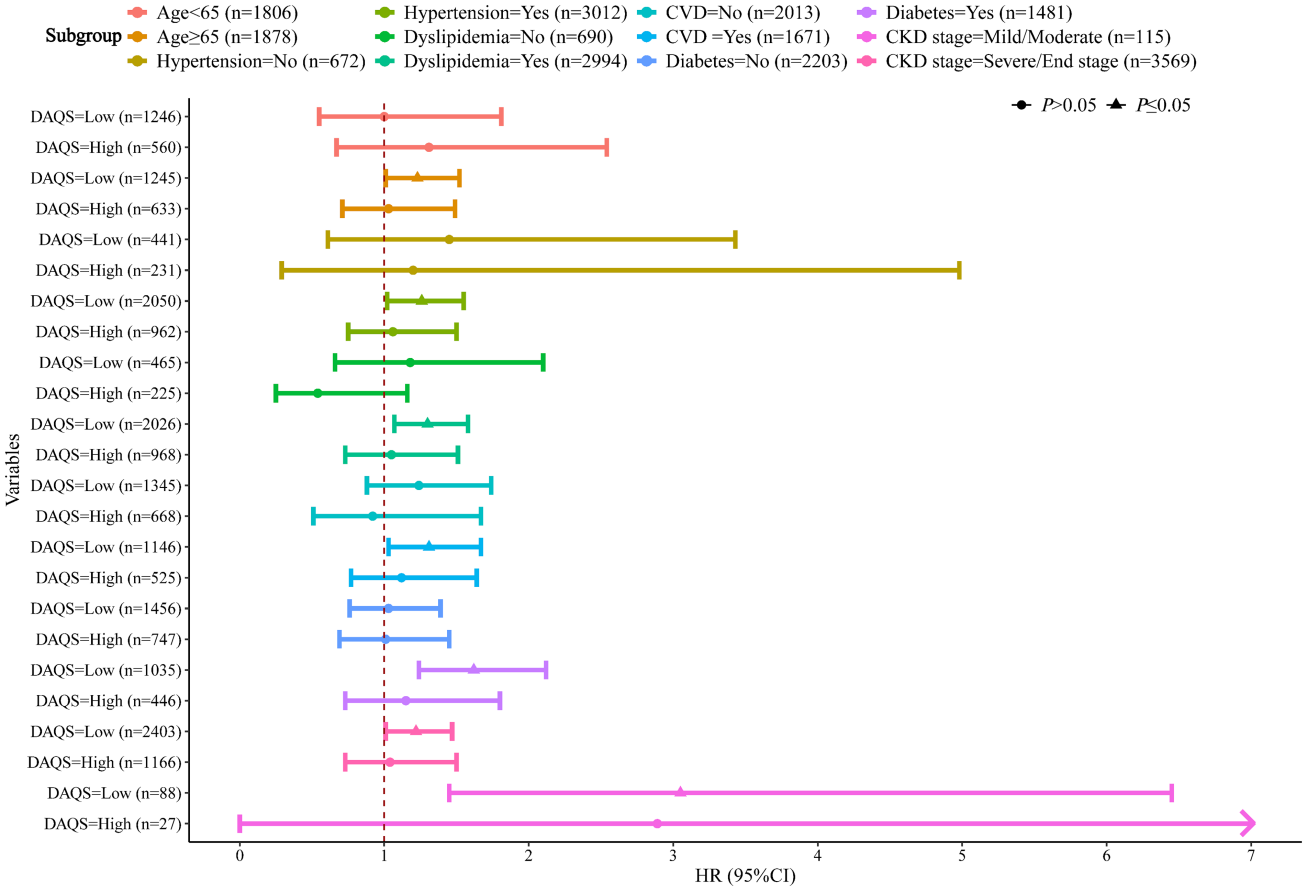
Association between dietary antioxidants intake and hyperuricemia related mortality by age, hypertension, dyslipidemia, CVD, diabetes and CKD stage among CKD patients. CVD, cardiovascular; CKD, chronic kidney disease.

In present study, the relationship was investigated between antioxidants intake and odds of hyperuricemia related mortality. After adjusted covariates, who found higher DAQS was associated with lower hyperuricemia related mortality in CKD patients. And subgroup analysis showed that this association was consistent across various subgroups, including individuals aged ≥65 years, individuals with hypertension, dyslipidemia, CVD and diabetes. These results suggested that higher antioxidants intake may facilitate the prognosis among CKD, and reduce odds of hyperuricemia related mortality in CKD patients (see Figure 2).

Moderate dietary antioxidants intake has shown potential benefit for CKD patients (13). UA was associated with adverse outcomes in patients with CKD (3, 6, 25). Excess UA could active OS, and antioxidants intake has a positive effect on hyperuricemia (26). By higher antioxidants intake, the deleterious synergistic effects of hyperuricemia and OS were counteracted, ultimately improving the prognosis of patients with CKD. In subgroup analysis, we also observed the association between low DAQS and hyperuricemia related mortality among elderly patients and patients with comorbidities. These results are consistent with previous studies (13, 14). It may be because these subgroups of individuals have higher levels of OS, and are more sensitive to the effects of exogenous dietary antioxidant intake. And our study shows a potential ameliorative effect of antioxidants intake on the odds of hyperuricemia related mortality among different CKD stages. OS was present in the early stages of CKD and progressed with worsening renal function, and was more severe in end-stage renal disease patients with hemodialysis (25).

The mechanisms underlying the ameliorative effect of dietary antioxidants intake on the risk of hyperuricemia related mortality in CKD patients could be explained through several pathways. Firstly, dietary antioxidants play a crucial role in counteracting OS by neutralizing ROS and protecting against cellular damage (25, 27). Antioxidants, such as vitamin A, C, and E, as well as minerals like zinc, selenium, and magnesium, scavenge free radicals and inhibit oxidative damage (13). Inadequate antioxidants intake may lead to a diminished antioxidant capacity, rendering CKD patients more susceptible to OS-induced damage. This imbalance between ROS production and antioxidant defense mechanisms can further exacerbate the pro-inflammatory state and endothelial dysfunction commonly observed in CKD (28). As a consequence, the increased OS may contribute to the progression of renal dysfunction, cardiovascular complications, and ultimately, mortality in CKD patients. Secondly, it is worth considering the interplay between UA and inflammation in CKD patients. Hyperuricemia has been associated with increased levels of pro-inflammatory cytokines, such as interleukin-6 (IL-6) and tumor necrosis factor-alpha (TNF-α) (29, 30). These inflammatory mediators can further stimulate UA production, creating a vicious cycle of inflammation and hyperuricemia (4). Inflammatory plays a pivotal role in the pathogenesis of CKD, promoting renal fibrosis, endothelial dysfunction, and CVD (31–33). Inflammatory milieu, combined with hyperuricemia, may have synergistic effects on CKD progression and mortality. Thirdly, it is important to consider the potential impact of dietary antioxidants on UA metabolism. Antioxidants, particularly vitamin C, have been shown to enhance UA excretion by stimulating renal urate transporters (34). Inadequate antioxidants intake may impair this excretion process, leading to UA accumulation and subsequently hyperuricemia. Additionally, antioxidants can inhibit xanthine oxidase (XO), the enzyme responsible for UA production, thereby reducing UA levels (35). Insufficient antioxidants intake may result in increased XO activity, promoting UA synthesis and exacerbating hyperuricemia. These mechanisms further support the association between lower antioxidants intake and hyperuricemia-related mortality in CKD patients.

The clinical importance of our findings lies in the potential for dietary interventions to modulate the risk of hyperuricemia related mortality in CKD patients. Encouraging patients to consume a diet rich in antioxidants, including fruits, vegetables, whole grains, and legumes, may offer a practical and cost-effective approach to improve prognosis in patients with high risk. Moreover, it highlights the importance of considering individual patient characteristics, such as age, comorbidities, and CKD stage, when tailoring dietary recommendations to optimize antioxidant intake.

There are several advantages of our study. The nationally representative sample and long-term follow-up afforded substantial power to detect association between dietary antioxidants intake and hyperuricemia related mortality risk. However, the study still has several limitations. First, dietary information was collected using a 24-h dietary recall, which may introduce recall bias and may not accurately represent usual dietary intake. Second, several potential factors may contribute to variability in serum UA levels, including dietary factors, medication use, renal function, genetic factors, and lifestyle factors such as alcohol consumption and physical activity. Additionally, laboratory methods for UA measurement can also contribute to variability. These suggested that potential factors should be considered in the interpretation of the study findings and their impact on the reliability of serum UA levels. Third, only all-cause mortality was investigated in the current study. As lower incidence of specific mortality, which made it’s impossible to investigate the association between dietary antioxidants intake and specific mortality.

## Conclusion

Antioxidants intake may have an ameliorative effect on the risk of hyperuricemia related mortality in CKD. Higher antioxidants intake reduced the risk of hyperuricemia related mortality in CKD patients. The findings highlight the clinical importance of promoting antioxidant-rich diets as part of the comprehensive management of CKD patients. Potential factors may contribute to variability in serum UA level, these should be considered for the explaining of the study. Future longitudinal and causal studies are required to validate our findings and explore the optimal strategies for implementing antioxidants interventions in CKD patients.

## Data availability statement

Publicly available datasets were analyzed in this study. This data can be found here: NHANES, https://www.cdc.gov/nchs/nhanes/.

## Ethics statement

The requirement of ethical approval was waived by the Affiliated Taizhou People's Hospital of Nanjing Medical University for the studies involving humans because the Affiliated Taizhou People's Hospital of Nanjing Medical University. The studies were conducted in accordance with the local legislation and institutional requirements. The participants provided their written informed consent to participate in this study.

## Author contributions

SS: Data curation, Formal analysis, Investigation, Methodology, Project administration, Supervision, Writing – original draft, Writing – review & editing. QF: Conceptualization, Data curation, Formal analysis, Investigation, Methodology, Project administration, Writing – review & editing.

## Footnotes

^1^ https://www.cdc.gov/nchs/data-linkage/mortality.htm
