# Supplementary material for: The association between dietary antioxidant quality score and uric acid related mortality in patients with chronic kidney disease
Source: Front Nutr. 2024 Jul 12;11:1408898. doi: 10.3389/fnut.2024.1408898 (PMC11275562; doi:10.3389/fnut.2024.1408898)
Supplement: Supplementary file 1 [file Table_1.docx]

Table S1 Sensitivity analysis of the data before and after manipulation of the missing values

| Variables | After (n=3684) | Before (n=3684) | Statistics | P |
| --- | --- | --- | --- | --- |
| PIR, Mean (S.E) | 2.63 (0.05) | 2.64 (0.06) | t=-1.46 | 0.147 |
| BMI, Mean (S.E) | 30.44 (0.19) | 30.44 (0.19) | t=-0.14 | 0.892 |
| WBC (1000 cells/uL), Mean (S.E) | 7.59 (0.06) | 7.59 (0.06) | t=-1.72 | 0.090 |
| Lymphocyte count, Mean (S.E) | 2.07 (0.03) | 2.07 (0.03) | t=-1.22 | 0.225 |
| Neutrophil count, Mean (S.E) | 4.65 (0.04) | 4.66 (0.04) | t=-1.79 | 0.078 |
| Platelet count, Mean (S.E) | 233.46 (1.84) | 233.45 (1.85) | t=0.74 | 0.461 |
| Hemoglobin, Mean (S.E) | 13.77 (0.05) | 13.78 (0.05) | t=-1.47 | 0.146 |
| ALP, Mean (S.E) | 74.17 (0.67) | 74.18 (0.67) | t=-1.00 | 0.323 |
| AST, Mean (S.E) | 26.43 (0.50) | 26.43 (0.50) | t=-0.87 | 0.389 |
| Lymphocyte count, Mean (S.E) | 30.44 (0.19) | 30.44 (0.19) | t=-1.46 | 0.147 |

S.E: standard error.
